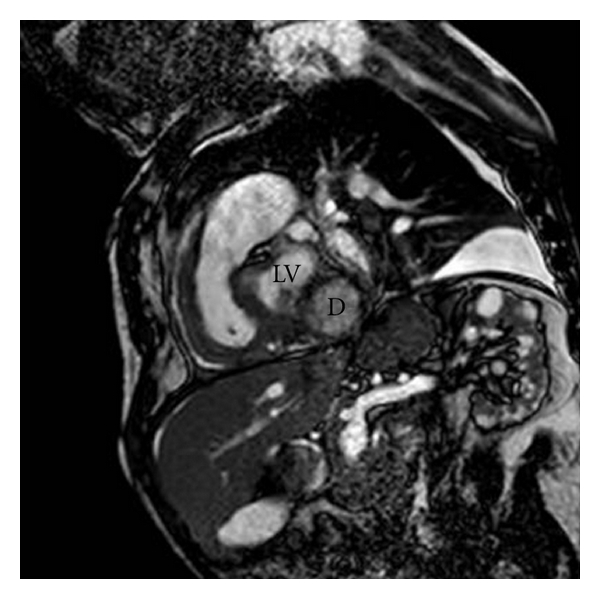# Left Ventricular “Diverticulum” in a Patient Affected by Galactosialidosis

**DOI:** 10.1155/2011/356056

**Published:** 2011-06-26

**Authors:** Alessandro Durante, Mariaemilia Traini, Roberto Spoladore

**Affiliations:** San Raffaele Scientific Institute and Vita-Salute San Raffaele University, Via Olgettina 60, 20132 Milan, Italy

## Abstract

We present the case of a 35-year-old man affected by the late juvenile form of galactosialidosis. He was known for a moderate pericardial effusion which remained unchanged in the last 12 months. Last follow-up transthoracic echocardiographic examination showed a bulging of the posterior and lateral wall of the left ventricle. This finding has never been described before in galactosialidosis.

## 1. Introduction

Galactosialidosis is a very rare form of lysosomal storage disease, whose prevalence is unknown. It is inherited in an autosomal recessive way. It is caused by a combined deficiency of beta-galactosidase and neuraminidase. The combined deficiency has been found to result from a defect in protective protein/cathepsin A, an intralysosomal protein which protects these enzymes from premature proteolytic processing [1]. 

Based on the age of onset and severity of the disease, galactosialidosis is divided into three clinical subtypes [2].

All patients have clinical manifestations typical of lysosomal storage disorder, such as coarse facies, cherry-red spots, vertebral changes, foam cells in the bone marrow, and vacuolated lymphocytes. The early infantile form is associated with fetal hydrops, edema, ascites, visceromegaly, skeletal dysplasia, and early death. The late infantile form of galactosialidosis shares some features with the early infantile form although the signs and symptoms are somewhat less severe and begin later in infancy. This form is characterized by short stature, dysostosis multiplex, heart valve problems, and hepatosplenomegaly. Other symptoms seen in some individuals with this type include intellectual disability, hearing loss, and a cherry-red spot. Children with this condition typically develop symptoms within the first year of life. The life expectancy of individuals with this type varies depending on the severity of symptoms. 

The juvenile/adult form of galactosialidosis has signs and symptoms that are somewhat different than those of the other two types. This form is distinguished by difficulty coordinating movements (ataxia), muscle twitches (myoclonus), seizures, and progressive intellectual disability. People with this form resembles those with the other forms in having dark red spots on the skin (angiokeratomas), abnormalities in the bones of the spine, “coarse” facial features, a cherry-red spot, vision loss, and hearing loss. The age at which symptoms begin to develop varies widely among affected individuals, but the average age is 16. This form is typically associated with a normal life expectancy.

## 2. Case Presentation

**Figure 1:**
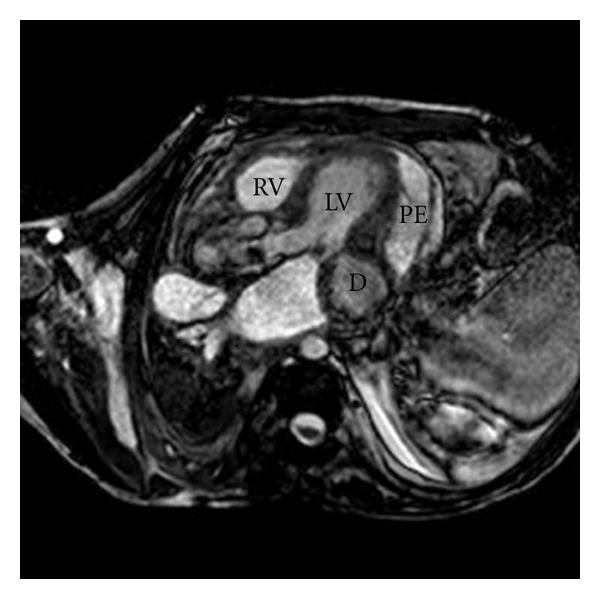


**Figure 2:**
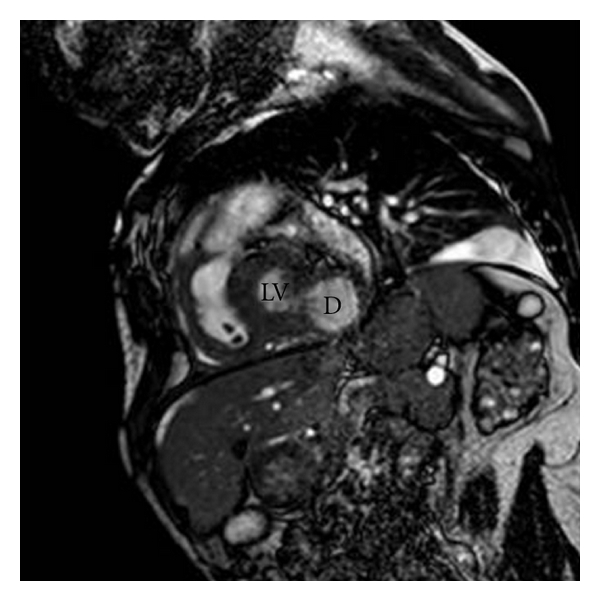


**Figure 3:**
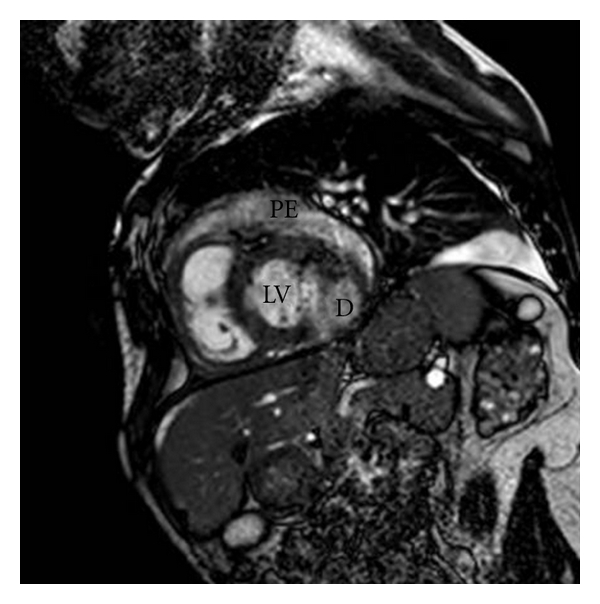


We present the case of a 35-year-old man affected by the late juvenile form of galactosialidosis. He presented mental and growth retardation, coarse facies, and chronic renal failure in haemodialitic treatment. He has been known for one year for pericardial effusion, which remained stable at periodic transthoracic echocardiographic examinations (TTEs). Last follow-up TTE showed a bulging of the posterior and lateral wall of the left ventricle which had a wide neck and a thin wall. It was not possible to discriminate between left ventricle aneurysm and pseudoaneurysm. The patient underwent a cardiac magnetic resonance (CMR). CMR showed a moderate circumferential pericardial effusion with fibrin exudates (Figures 1 and 3). The medium segment of the left ventricular lateral wall was thickened and vacuolated. It continued in the posterior and lateral bulging, where the wall was thinner (about 4-5 mm) (Figures 1–4). The visceral layer of the pericardium was not discontinued and contained the whole bulging (Figure 1). The maximum diameter of the bulging was 30 mm (Figure 3), and the neck measured 10 mm at the end-diastole, while it was much smaller at the end-systole (Figures 1 and 2). The whole wall of the bulging showed systolic thickening, except for a very small area where the end-diastolic thickness was the most reduced. The strange appearance of the “aneurismatic” formation resembles that of intestinal diverticulum, and we named it “left ventricular diverticulum” (see video 1 at Supplementary Material available online at doi: 10.1155/2011/356056). The decision not to refer the patient for cardiac surgery and bulging excision was thoroughly discussed and agreed, eventually due to the high surgical risk. The patient underwent followup with TTE at 1 and 6 months and CMR at 12 months. Their findings were unchanged. Due to the stability over time of the pericardial effusion we decided not to refer the patient for pericardial drainage.

## 3. Discussion

The bulging has a wide neck, which is typical of aneurysm. In the medium segment of the left ventricle, where it originates from the posterior and lateral walls, myocardial tissue is thickened and vacuolated. This appearance could be related to the presence of vacuolated cells, which is well described in other organs in patients affected by galactosialidosis [3].

The presence of vacuolated cells could make the wall of the diverticulum less resistant, which could be the cause of its expansion under the left ventricular pressure. This hypothesis could also explain the presence of a thinner wall, which is derived from the stretched tissue with vacuolated cells. The variability in neck diameter during systole and diastole indicates the muscular origin of the diverticulum.

The typical cardiac finding in galactosialidosis is valve involvement. Our patient showed only mildly myxomatous valves without any significant valve dysfunction. 

Pericardial effusion is not described in the literature, but it could be related to the previously described peritoneal effusion. 

The more striking finding is the presence of the cardiac bulging, which remained stable in a 12-month followup.

## 4. Conclusion

To our knowledge this is the first report of a cardiac bulging in galactosialidosis. 

This case is only hypothesis originating since we did not performen domyocardial biopsy taking in account the high risk of free wall rupture of the left ventricle, but we will follow up the patient with a CMR with spectroscopy.

## Supplementary Material

The video shows short axis cine MR from apex to base with evidence of the left ventricular diverticulum.

## Figures and Tables

**Figure 4:**